# Trends in Food Insecurity and SNAP Participation among Immigrant Families of U.S.-Born Young Children

**DOI:** 10.3390/children6040055

**Published:** 2019-04-04

**Authors:** Allison Bovell-Ammon, Stephanie Ettinger de Cuba, Sharon Coleman, Nayab Ahmad, Maureen M. Black, Deborah A. Frank, Eduardo Ochoa, Diana B. Cutts

**Affiliations:** 1 Boston Medical Center, Boston, MA 02118, USA; nayab.ahmad@bmc.org (N.A.); dafrank@bu.edu (D.A.F.); 2 Boston University School of Medicine, Boston, MA 02118, USA; sedc@bu.edu; 3 Boston University School of Public Health, Boston, MA 02118, USA; Sharcole2@gmail.com; 4 University of Maryland School of Medicine; Baltimore, MD 21201, USA; mblack@peds.umaryland.edu; 5 Research Triangle Institute, Research Triangle Park, NC 12194, USA; 6 University of Arkansas for Medical Sciences, Little Rock, AR 72205, USA; ochoaeduardor@uams.edu; 7 Hennepin County Medical Center, Minneapolis, MN 55415, USA; diana.cutts@hcmed.org

**Keywords:** immigrant families, food insecurity, supplemental nutrition assistance program

## Abstract

Immigrant families are known to be at higher risk of food insecurity compared to non-immigrant families. Documented immigrants in the U.S. <5 years are ineligible for the Supplemental Nutrition Assistance Program (SNAP). Immigration enforcement, anti-immigrant rhetoric, and policies negatively targeting immigrants have increased in recent years. Anecdotal reports suggest immigrant families forgo assistance, even if eligible, related to fear of deportation or future ineligibility for citizenship. In the period of January 2007–June 2018, 37,570 caregivers of young children (ages 0–4) were interviewed in emergency rooms and primary care clinics in Boston, Baltimore, Philadelphia, Minneapolis, and Little Rock. Food insecurity was measured using the U.S. Department of Agriculture’s Food Security Survey Module. Overall, 21.4% of mothers were immigrants, including 3.8% in the U.S. <5 years (“<5 years”) and 17.64% ≥ 5 years (“5+ years”). SNAP participation among <5 years families increased in the period of 2007–2017 to 43% and declined in the first half of 2018 to 34.8%. For 5+ years families, SNAP participation increased to 44.7% in 2017 and decreased to 42.7% in 2018. SNAP decreases occurred concurrently with rising child food insecurity. Employment increased 2016–2018 among U.S.-born families and was stable among immigrant families. After steady increases in the prior 10 years, SNAP participation decreased in all immigrant families in 2018, but most markedly in more recent immigrants, while employment rates were unchanged.

## 1. Introduction

One-quarter of children in the United States (U.S.) under age 5 have at least one immigrant parent, with 93% of these children born in the U.S. [1]. Previous research has shown that infants and toddlers in low-income families with immigrant mothers are more likely to be born at a healthy weight, to be breastfed, to live in a two-parent home, and to have mothers who do not use tobacco, compared to children in low-income families with U.S.-born mothers [2]. Immigrant families, however, compared to non-immigrant families, disproportionately experience food insecurity, struggle to afford housing costs, and lack access to health care—all factors associated with adverse health outcomes [3,4].

Food insecurity, even if experienced at mild levels or temporarily, is associated with poor physical and mental health for children and adults regardless of nativity or immigration status [5,6,7,8,9,10,11]. As the severity of food insecurity increases to affect the quality and quantity of children’s food, the health impacts of food insecurity on child health also worsen [12]. The Supplemental Nutrition Assistance Program (SNAP), the largest nutrition program in the U.S., is strongly associated with improved food security and positive health outcomes from the pre-natal period through early childhood and into adulthood [13,14].

SNAP is a means-test entitlement program that is available to all citizens and legally authorized families and individuals with incomes low enough to meet eligibility criteria. Families are often made aware of the program through community-based resource connections, and information about the program is widely available. In 2017, approximately two-thirds of people participating in SNAP were children, seniors, or persons with disabilities, and the average household income for SNAP participants was 63% of the U.S. federal poverty line [15]. Families and individuals participating in SNAP receive a monthly allotment of funds that are restricted for the sole purpose of purchasing uncooked foods to be prepared at home. SNAP cannot be used to purchase hot foods, alcoholic beverages, vitamins, cigarettes, household supplies, or other non-food items. These benefits are issued monthly on an electronic benefit transfer (EBT) card that the participant is able to use at authorized food retailers. In addition to reducing food insecurity, SNAP also promotes better nutrition. Every state in the U.S. operates SNAP nutrition education programming designed to teach participants about the benefits of healthy eating [16].

While all SNAP participants live in households with low incomes and therefore have higher rates of food insecurity than higher income households, several studies have documented the program’s effectiveness in reducing food insecurity across levels of severity [13,14,17]. SNAP is also a countercyclical program, designed to expand during recessions when unemployment rates are high—as it did during the recent Great Recession, which began in December 2007 and officially ended in June 2009—and contract when unemployment rates are lower. Because of the countercyclical nature of SNAP, it is sensitive to trends in employment.

A large body of evidence documents the link between SNAP and child health. Mothers who participate in SNAP during pregnancy are more likely to have healthier babies compared to SNAP-eligible non-participants [18]. Young children in SNAP-participating families are less likely to be hospitalized, underweight, or at risk of developmental delays compared to SNAP-eligible non-participating families [19]. SNAP has also been shown to reduce food insecurity and poor health outcomes among children of all ages [20,21,22]. Even though SNAP effectively reduces food insecurity and improves health, it is underutilized, particularly by immigrant families. Federal regulations specify that documented immigrant adults who have been in the U.S. for less than five years are ineligible for SNAP, even if they meet all other eligibility criteria [23]. This is commonly known as the five-year bar. Although many families’ U.S. citizen children may qualify for SNAP, research demonstrates that when parents are ineligible for assistance, their eligible children are less likely to participate in assistance programs [24]. Consequently, children of non-citizen parents are less likely to participate in SNAP. Because the benefits, when accessed, are often for the children only, mixed immigration status households have lower levels of SNAP benefits per household member when they do participate in SNAP and are at greater risk of food insecurity compared to households where parents and children are all citizens [25].

Over the past ten years, and particularly since 2016, increased immigration law enforcement, threatening anti-immigrant rhetoric, and public policy proposals that target immigrant families, including those that penalize immigrants for participating in assistance programs, have increased [26,27]. Anecdotal reports suggest that immigrant families may be forgoing participation in nutrition assistance and other federal assistance programs, even if eligible, due to fear of deportation or the negative effect of participation on their future U.S. immigration status [28,29].

We are unaware of any research that has systematically examined quantitative data comparing time trends in food security and SNAP participation among immigrant and non-immigrant families. This study aims to first document 10-year trends in household and child food security status and SNAP participation among families with young children disaggregated by maternal nativity and, for mothers born outside of the U.S., tenure of U.S. residence. The secondary aim of this study, given the changes in the policy environment from 2016 to 2018, sought to understand trends in food security status, SNAP participation, employment, and demographic differences across these years. Changes in household employment among immigrant and non-immigrant families, which may explain changes in SNAP participation and food insecurity rates, were also examined.

## 2. Methods

Data come from the ongoing Children’s HealthWatch study, a multisite cross-sectional study investigating associations between economic hardships, participation in assistance programs, and the health of young children and their families [30]. Caregivers of children under 48 months were recruited for survey participation by trained research assistants during their child’s primary care appointment or emergency department visit in five U.S. cities (Baltimore, MA; Boston, MA; Minneapolis, MN; Little Rock, AR; Philadelphia, PA). Data for this study were collected between January 2007 and June 2018, a period encompassing the Great Recession and economic recovery. As previously published [31], eligibility included fluency in English, Spanish, or Somali (Minneapolis only), state residency, and knowledge of the child’s household. Caregivers of critically ill or injured children were not approached, nor were those interviewed within the previous six months. Research assistants administered interviews to caregivers verbally face-to-face in private settings after gaining informed consent. Institutional review board approval was obtained at each site prior to data collection and was renewed annually.

**Figure 1:**
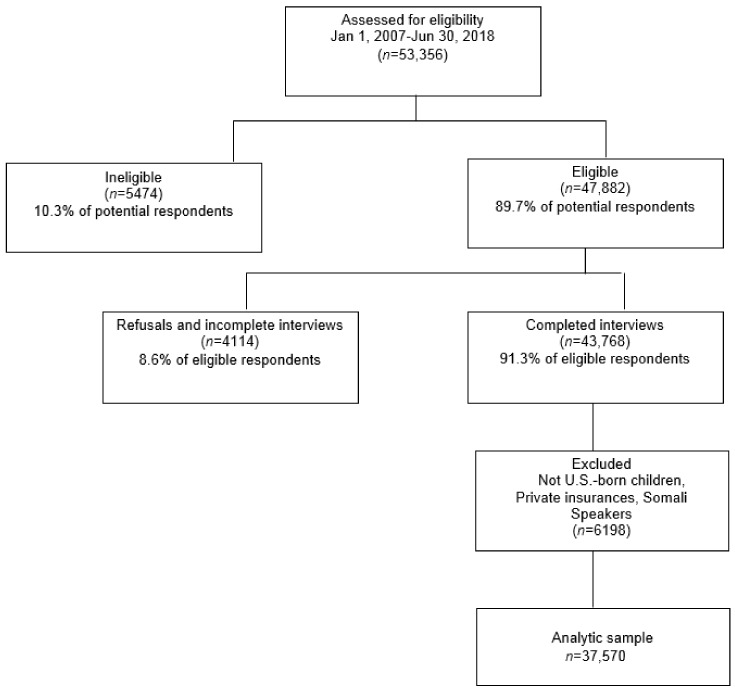
Description of analytic sample.

Of 53,356 caregivers approached between January 2007 and June 2018, 5474 (10.3%) were ineligible for the study, and 4114 (8.6%) refused or were unable to complete the interview. To ensure that the sample included only families with some members likely to be eligible for SNAP, the sample was limited to children born in the U.S. with public or no health insurance. Of caregiver/child dyads who completed the interview, 354 (<1%) children born outside of the U.S. and 4342 (9.98%) children with private health insurance were excluded. Additionally, the sample excluded caregivers who completed the interview in Somali (*n* = 168), given the unique circumstances of Somali refugees in the U.S., who are more likely to be eligible for SNAP than other immigrant populations and to whom the five-year bar does not apply. The final analytic sample was 37,570 (Figure 1).

### 2.1. Independent Variables

#### 2.1.1. Demographics

Caregivers reported the mother’s age and race/ethnicity, their educational attainment, and their employment status. Child age was abstracted from medical records.

Mother’s nativity and tenure in the U.S.: Caregivers were asked the birthplace of the biological mother and, if born outside of the U.S., the year the biological mother moved to the U.S. Of the caregivers interviewed, 93.7% were biological mothers. The sample was divided into three groups by nativity and tenure in the U.S.: (1) mothers born in the U.S. (U.S.-born group); (2) mothers born outside of the U.S. residing in the U.S. for five or more years (5+ years group); and (3) mothers born outside of the U.S. residing in the U.S. for less than five years, reflecting SNAP’s five-year residency requirement (<5 years group).

#### 2.1.2. Employment

Caregivers reported the number of employed members in the household. For this analysis, the variable was coded as any household employment vs. no household employment. Additionally, employment trends focused on the most recent years across the three groups—2016 through 2018. These years were selected in order to detect whether any change in food security or SNAP status in this period was plausibly related to increasing employment.

### 2.2. Dependent Variables

#### 2.2.1. Food Insecurity

Household and child food insecurity were measured using the U.S. Household Food Security Survey Module (HFSSM). This survey module consists of 10 household-focused questions and eight child-specific questions assessing the previous 12 months. The HFSSM is the gold standard in the U.S. for assessing food insecurity. Households are considered food insecure if they report they are were unable to consistently afford enough food for all household members to lead active, healthy lives, and if this condition was a result of constrained resources. These analyses identified two levels of food insecurity: (1) household food insecurity (HFI)—three or more household-focused questions endorsed as sometimes true or often true vs. never true, but none on the child-specific scale; and (2) child food insecurity (CFI)—two or more child-specific questions endorsed as sometimes true or often true vs. never true.

#### 2.2.2. SNAP Participation

Caregivers were asked whether their household participated in SNAP at the time of the interview.

### 2.3. Analysis

To examine the prevalence of household food insecurity, child food insecurity, and participation in SNAP stratified by maternal nativity and tenure in the U.S., we examined changes in each variable independently for each year over the study period, in addition to the 6 months from January–June 2018. In a secondary analysis, we analyzed changes in employment status between 2016–2018 stratified by maternal nativity and tenure in the U.S. Prevalence rates were compared across years through chi-square tests using a significance level of 0.05. All analyses were conducted using SAS software (version 9.3; SAS Institute, Cary, NC, U.S.).

## 3. Results

Overall, 78.6% of the households had U.S.-born mothers, 17.6% had immigrant mothers in the U.S. ≥5 years, and 3.8% had immigrant mothers in the U.S. <5 years.

**Figure 2:**
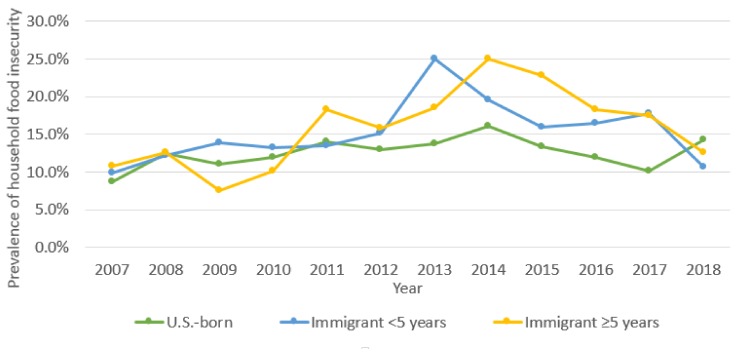
Trends in household food insecurity 2007–2018 by mother’s place of birth.

The primary analysis found household food insecurity among all groups increased over the study period. Household food insecurity among the U.S.-born group increased from 8.7% in 2007 to 14.3% in the first half of 2018, reaching its highest point in 2014, with a prevalence of 16.1% (*p* < 0.0001). The 5+ years group experienced an increase in household food insecurity from 10.8% in 2007 to 25.0% in 2014, then a steady decrease to 12.6% by the first half of 2018 (*p* < 0.0001). Household food insecurity among the <5 years group increased from 9.9% in 2007 to 25.0% in 2013 and then declined to 10.6% in the first six months of 2018 (*p* = 0.04) (Figure 2).

**Figure 3:**
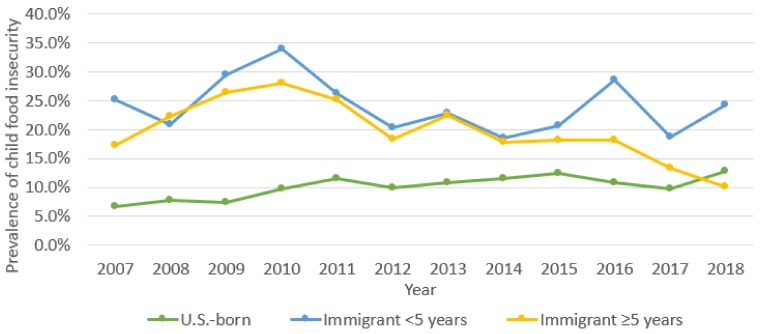
Trends in child food insecurity 2007–2018 by mother’s place of birth.

Child food insecurity rates fluctuated among groups across the study period. Increasing from 6.7% in 2007 among the U.S.-born group, the prevalence in this group peaked at 12.7% in the first six months of 2018 (*p* < 0.0001). Child food insecurity rates for the 5+ years group increased from 17.2% in 2007 to 28.0% in 2010, then declined to 10.1% in 2018 (*p* < 0.0001). Child food insecurity was consistently highest among the <5 years group. In 2007, rates of child food insecurity were 25.2% among this group, increased to 33.9% in 2010, and then declined to 24.2% in the first half of 2018. The highest rate of child food insecurity among the <5 years group was in 2010 during the immediate aftermath of the recession, with a prevalence of 33.9%, declining over the next six years to 18.7% in 2017, though increasing again to 28.6% (*p* = 0.035) in the first half of 2018 (Figure 3).

**Figure 4:**
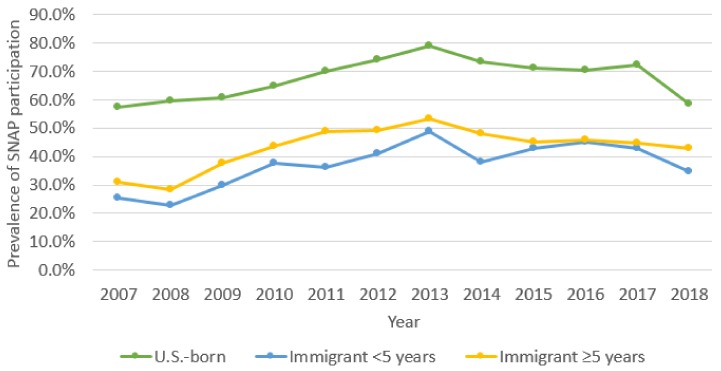
Trends in SNAP participation 2007–2018 by mother’s place of birth.

SNAP participation varied across the groups and study years. Among the U.S.-born group, rates of SNAP participation increased from 57.2% in 2007 to 78.8% in 2013 and then steadily declined. SNAP participation among the 5+ years group was 30.8% in 2007, then rose to a high of 53.3% in 2013 before steadily decreasing to 42.7% in the first half of 2018. In the <5 years group, SNAP participation increased from 25.4% in 2007, to 48.9% by 2013, decreased to 43.0% in 2017 and then further decreased to 34.8% in the first half of 2018. All differences are significant at *p* < 0.0001 (Figure 4).

The secondary analysis found differences in demographics and employment, which varied across groups from 2016 to 2018. Among U.S.-born mothers, there were demographic changes in the sample between 2016 and 2018. In 2016, 17.1% of mothers were White compared to 29.6% in 2018. There were also fewer Hispanic mothers and Black mothers in 2018 compared to 2016 (21.8% vs. 20.2% and 58.3% vs. 46.4% respectively) (*p* < 0.0001). The average age of U.S.-born mothers in 2016 was 26.6 (SD 5.3) and 27.3 (SD 5.5) in 2018 (*p* = 0.0004). The average age of the child was 19.2 months (SD 13.5) and did not vary across years. Caregivers in these families also had higher rates of education in 2018 than 2016, with 46.3% reporting having completed education beyond high school compared to 39.2% in 2016 (*p* < 0.0001); additionally, there were higher rates of being married/partnered in 2018 compared to 2016 (26.1% vs. 19%, respectively).

Among families in the 5+ years group, there were no demographic differences from 2016 to 2018. Across all three years, 73.5% of mothers were Hispanic, 21.2% Black, and 0.9% White. On average, mothers were 31.8 years old (SD 6.5) and children were 20.1 months (SD 14.3) old. One-quarter of caregivers (24.9%) had education beyond high school, and 43.5% were married or partnered. Among the <5 years group, on average from 2016 to 2018, 77.9% of mothers were Hispanic, 18.7% Black, and 0.8% White, which did not vary across years. The mean age of mothers was 28.7 (SD 6.5) years old across all three years. Child’s age was the only demographic variable that changed significantly from 2016 to 2018 (12.9 (SD 11.9) vs. 18.3 (SD 12.9) months, respectively). Within this group, 34.6% of caregivers reported education beyond high school, and 40.2% were married or partnered, which did not vary by year (Table 1).

Analysis of employment trends from 2016–2018 also showed differences in employment rates across groups. Among U.S.-born mothers, household employment status increased from 78.3% in 2016 to 81.4% in 2018 (*p* = 0.0014). Household employment rates were, on average, 91% in the 5+ years group and 85.9% among families in the <5 years group, with no significant changes from 2016–2018 (Table 1).

## 4. Discussion

During the Great Recession from 2007 to 2009, food insecurity increased for families with young children across all three groups. Families with immigrant mothers, in particular, had higher rates of household and child food insecurity during the height of the recession and a slower recovery than families with U.S.-born mothers. SNAP participation also increased across all groups between 2007 and 2013 during the Great Recession and its aftermath and then began to decline However, among families with U.S.-born mothers and immigrant mothers in the U.S. <5 years, there was a sharp decrease in SNAP participation between 2017 and the first half of 2018. However, small cell sizes for the first 6 months of 2018 should be interpreted with caution.

While an improving economy with higher employment rates might be a plausible explanation for this sharp decrease in participation in SNAP, employment trends varied only for the families with U.S.-born mothers while remaining constant for families with immigrant mothers in the U.S. <5 years. The decrease in SNAP participation occurring concurrently with both an increase in employment and an increase in food insecurity among families with U.S.-born mothers may reflect a previously documented phenomenon where families whose SNAP benefits are cut off due to increased earnings experience a net loss of family resources placing them at higher risk of food insecurity [32]. The consistency of employment across 2016–2018 occurring concurrently with a decline in SNAP benefits among families with immigrant mothers who have resided in the U.S. <5 years, however, suggests that other factors may be contributing to this trend. The decline in participation among these families may be reflective of recent anecdotal reports suggesting that immigrant families are dis-enrolling or declining to enroll in federal assistance programs, including SNAP, out of fear of deportation or deleterious impacts on their future U.S. immigration status [29,33,34]. Other reasons for the decline in participation may be associated with this trend. Further research, however, is needed to discern the cause of the decrease. Qualitative methods that provide opportunities for immigrant mothers to respond to open-ended questions pertaining to their experiences in the U.S. and the reasons why they choose to participate or not participate in federal assistance programs such as SNAP may offer greater insights into the trends identified through this study. Research utilizing administrative data may also be able to examine nationally representative trends and potentially uncover other reasons for declining participating, such as disproportionate terminations or denials.

Several limitations of this analysis should be considered. The data come from cross-sectional sampling and therefore demonstrate associations, not causation. Due to only 6 months of available data for 2018, the cell sizes for this time period are small and therefore should be interpreted cautiously. All outcomes were self-reported, which creates a potential for bias in food security status and over- or underreporting of SNAP participation. Given that the current study includes only unadjusted outcomes, more research is necessary to examine associations adjusted for contextual factors that may relate to food security or SNAP participation. Further, the immigration status for the mothers, which impacts eligibility for SNAP, is unknown. The current policy context, however, makes these preliminary findings timely, and provides important evidence for ongoing policy discussions as well as directions for future research.

Policy proposals, such as the recent regulatory proposal to change the definition of public charge, may have contributed to this trend. Beginning in February of 2017, the federal administration began discussing changes to public charge, which is a term used by U.S. immigration officials to refer to persons who are considered primarily dependent on the government for subsistence. Immigrants subject to this consideration who are found to be or likely to become a public charge may be denied admission to the U.S. or denied adjustment to legal permanent resident status. To date, public charge determination has been limited to receipt of public cash assistance or institutionalization for long-term care at the government’s expense.

Data in this study suggest a declining trend in SNAP participation among immigrant families, even as their employment remains constant and child food insecurity continues at a rate higher than the U.S.-born population. If the definition of public charge were expanded, as currently proposed by the present administration, to include participation in SNAP and potentially other supports like housing subsidies and Medicaid (public health insurance), rates of food insecurity among citizen children under the age of four years are likely to increase, along with associated health consequences [35,36]. Anecdotal stories from physicians, social service providers, and members of the community already describe fear among immigrant families related to participation in SNAP. A change to public charge could sharply increase this phenomenon in the short term.

Given the immediate and long-term health implications of food insecurity, especially child food insecurity [12], policy proposals that change public charge determination rules or impede SNAP participation among immigrant families of U.S. citizen infants and toddlers could have long-term negative consequences on public health and the health care system [37,38]. Beyond public policy change, it is important to increase education efforts among non-governmental, community-based organizations working with immigrant communities to inform immigrant families of their eligibility for SNAP and provide resources to local organizations that support enrollment in SNAP and other programs. In addition, ensuring data confidentiality for those applying for benefits, eliminating hostile anti-immigrant rhetoric in national discourse, and reducing barriers to nutrition assistance for families with low incomes regardless of parental nativity or immigration status [39] may benefit the health, growth, and development of the youngest citizens of the U.S. Future research is necessary to examine the sequelae of health outcomes associated with the trends documented in this study and identify potential solutions to remediate these trends. Further, as leaders in other countries outside of the U.S. propose policies that negatively target immigrants, research would be important to discern the potential ripple effects on the health of children and their families in those settings.

## 5. Conclusions

Over the last ten years, household food insecurity doubled for families with recently arrived immigrant mothers and their U.S.-born children while rates of child food insecurity remained alarmingly high. SNAP participation for these families decreased between 2017 and the first half of 2018, despite a lack of change in household employment status. These trends may be reflective of anecdotal reports in recent years that immigrant families fear participation in health-promoting nutrition assistance programs for which they may be eligible, including SNAP, because of fears of deportation or effects on their future immigration status. Policies that increase, rather than decrease, support for immigrant families with infants and toddlers may be necessary for reversing these trends that threaten the health and development of young children and their families.

## Figures and Tables

**Table 1:** Demographics for 2016 to 2018 by mother’s nativity and tenure in the U.S.

Question	Response	Overall	2016	2017	2018	*p*-Value
**U.S.-Born Mothers**						
Mother Age	*N*	5132	2266	1538	1328	0.0004
	Mean (Std Dev)	26.8 (5.5)	26.6 (5.3)	26.8 (5.6)	27.3 (5.5)	
	*N*	5189	2287	1555	1347	
Child Age (Months)	Mean (Std Dev)	19.2 (13.5)	19.4 (13.7)	18.6 (13.3)	19.6 (13.4)	0.1207
Mother’s Race/Ethnicity	Hispanic	1208 (23.6%)	492 (21.8%)	446 (29.2%)	270 (20.2%)	<0.0001
	Black|Non-Hispanic	2770 (54.1%)	1314 (58.3%)	837 (54.8%)	619 (46.4%)	
	White|Non-Hispanic	981 (19.2%)	386 (17.1%)	200 (13.1%)	395 (29.6%)	
	Other	158 (3.1%)	63 (2.8%)	45 (2.9%)	50 (3.7%)	
Caregiver Married/Partnered	Yes	1036 (20.0%)	434 (19.0%)	251 (16.2%)	351 (26.1%)	<0.0001
Caregiver Education	Less than high school	853 (16.5%)	382 (16.7%)	275 (17.7%)	196 (14.6%)	<0.0001
	High school	2231 (43.0%)	1009 (44.1%)	694 (44.7%)	528 (39.2%)	
	More than high school	2101 (40.5%)	895 (39.2%)	583 (37.6%)	623 (46.3%)	
Any Employment in household	Yes	4019 (78.4%)	1773 (78.3%)	1160 (75.8%)	1086 (81.4%)	0.0014
**Immigrant Mothers ≥5 Years ^a^**						
Mother Age	*N*	1297	530	481	286	
	Mean (Std Dev)	31.8 (6.5)	31.6 (6.4)	31.7 (6.4)	32.1 (6.7)	0.5691
Child Age (Months)	*N*	1297	530	481	286	
	Mean (Std Dev)	20.1 (14.3)	20.7 (14.6)	19.6 (14.0)	19.8 (14.4)	0.3960
Mother Ethnicity	Hispanic	950 (73.5%)	398 (75.2%)	352 (73.8%)	200 (69.9%)	0.7082
	Black|Non-Hispanic	287 (22.2%)	112 (21.2%)	104 (21.8%)	71 (24.8%)	
	White|Non-Hispanic	12 (0.9%)	3 (0.6%)	5 (1.0%)	4 (1.4%)	
	Other	43 (3.3%)	16 (3.0%)	16 (3.4%)	11 (3.8%)	
Caregiver Married/Partnered	Yes	563 (43.5%)	230 (43.6%)	208 (43.2%)	125 (43.9%)	0.9858
Caregiver Education	Less than high school	485 (37.4%)	202 (38.1%)	191 (39.7%)	92 (32.2%)	0.1215
	High school	489 (37.7%)	207 (39.1%)	164 (34.1%)	118 (41.3%)	
	More than high school	91 (34.6%)	29 (31.9%)	40 (37.7%)	22 (33.3%)	
Any Employment in Household	Yes	1175 (91.0%)	474 (89.8%)	439 (92.0%)	262 (91.6%)	0.4222
**Immigrant Mothers <5 Years ^b^**						
Mother Age	*N*	264	91	107	66	
	Mean (Std Dev)	28.7 (6.5)	27.9 (6.3)	30.0 (6.6)	27.8 (6.3)	0.0293
Child Age (Months)	*N*	264	91	107	66	
	Mean (Std Dev)	14.4 (12.1)	12.9 (11.9)	13.2 (11.3)	18.3 (12.9)	0.0095
Mother Ethnicity	Hispanic	204 (77.9%)	72 (80.0%)	85 (79.4%)	47 (72.3%)	0.4806
	Black|Non-Hispanic	49 (18.7%)	13 (14.4%)	19 (17.8%)	17 (26.2%)	
	White|Non-Hispanic	2 (0.8%)	1 (1.1%)	1 (0.9%)	0 (0.0%)	
	Other	7 (2.7%)	4 (4.4%)	2 (1.9%)	1 (1.5%)	
Caregiver Married/Partnered	Yes	106 (40.2%)	39 (42.9%)	44 (41.1%)	23 (34.8%)	0.5794
Caregiver Education	Less than high school	91 (34.6%)	32 (35.2%)	36 (34.0%)	23 (34.8%)	0.9208
	High school	81 (30.8%)	30 (33.0%)	30 (28.3%)	21 (31.8%)	
	More than high school	91 (34.6%)	29 (31.9%)	40 (37.7%)	22 (33.3%)	
Any Employment in Household	Yes	225 (85.9%)	79 (86.8%)	89 (84.8%)	57 (86.4%)	0.9111

^a^ Immigrant Mothers ≥5 years: Families with mothers who immigrated to the U.S. more than or equal to five years ago. ^b^ Immigrant Mothers <5 years: Families with mothers who immigrated to the U.S. less than five years ago.
